# Knockdown of LRP/LR Induces Apoptosis in Breast and Oesophageal Cancer Cells

**DOI:** 10.1371/journal.pone.0139584

**Published:** 2015-10-01

**Authors:** Thandokuhle Khumalo, Eloise Ferreira, Katarina Jovanovic, Rob B. Veale, Stefan F. T. Weiss

**Affiliations:** School of Molecular and Cell Biology, University of the Witwatersrand, Private Bag 3, Wits 2050, Johannesburg, The Republic of South Africa (RSA); The Scripps Research Institute Scripps Florida, UNITED STATES

## Abstract

Cancer is a global burden due to high incidence and mortality rates and is ranked the second most diagnosed disease amongst non-communicable diseases in South Africa. A high expression level of the 37kDa/67kDa laminin receptor (LRP/LR) is one characteristic of cancer cells. This receptor is implicated in the pathogenesis of cancer cells by supporting tumor angiogenesis, metastasis and especially for this study, the evasion of apoptosis. In the current study, the role of LRP/LR on cellular viability of breast MCF-7, MDA-MB 231 and WHCO1 oesophageal cancer cells was investigated. Western blot analysis revealed that total LRP expression levels of MCF-7, MDA-MB 231 and WHCO1 were significantly downregulated by targeting LRP mRNA using siRNA-LAMR1. This knockdown of LRP/LR resulted in a significant decrease of viability in the breast and oesophageal cancer cells as determined by an MTT assay. Transfection of MDA-MB 231 cells with esiRNA-RPSA directed against a different region of the LRP mRNA had similar effects on LRP/LR expression and cell viability compared to siRNA-LAMR1, excluding an off-target effect of siRNA-LAMR1. This reduction in cellular viability is as a consequence of apoptosis induction as indicated by the exposure of the phosphatidylserine protein on the surface of breast MCF-7, MDA-MB 231 and oesophageal WHCO1 cancer cells, respectively, detected by an Annexin-V/FITC assay as well as nuclear morphological changes observed post-staining with Hoechst. These observations indicate that LRP/LR is crucial for the maintenance of cellular viability of breast and oesophageal cancer cells and recommend siRNA technology targeting LRP expression as a possible novel alternative technique for breast and oesophageal cancer treatment.

## Introduction

14.1 million cancer cases were diagnosed and 8.2 million deaths were attributed to cancer in the year 2012, with a majority of deaths occurring in developing countries such as South Africa (World Health Organization (GLOBOCAN 2012). In South Africa and worldwide, breast cancer is the most common cancer in woman and oesophageal cancer the 8^th^ most common cancer in both sexes worldwide [1]. Cancer is initiated by the accumulation of multiple mutations that result in the dysregulation of cellular homeostasis due to uncontrolled proliferation and lack of apoptosis of these genomically unstable/toxic cells[2]. Transformation to a cancerous cell is not an effortless transition but is a multistep process that may be due to alterations in the million processes that occur in a cell daily and the most fundamental alterations have been termed as the hallmarks of cancer by Hanahan and Weinberg[2]. These include tissue invasion and metastasis, insensitivity to growth inhibitors, self-sufficiency in growth signals, limitless replicative potential, sustained angiogenesis and the evasion of apoptosis[2]. Another prominent feature of tumors is the altered expression of oncogenes, tumor suppressor genes or receptors for sustained growth and progression; and one outstanding characteristic, is the overexpression of the 37-kDa/67-kDa laminin receptor precursor/ laminin receptor (LRP/LR)[2–4].

The 37-kDa/67-kDa laminin receptor (LRP/LR) is a non-integrin cell surface glycoprotein that interacts with numerous extracellular matrix proteins and more importantly its’ primary ligand, laminin-1[5]. Since its discovery in 1983 numerous subcellular localizations and multiple functions have been described, both physiological and pathological [4, 6–9]. LRP/LR also localises in the nucleus and the cytosol, is involved in the maintenance of nuclear structures and translational processes, respectively [10–15]. In addition to laminin-1, LRP/LR has several functions by acting as a receptor for other molecules at the cell membrane, acting as a receptor for carbohydrates, elastin[16] and also poses morbid effects to the cells by facilitating the internalization of viruses[17–19], infectious and non-infectious prion proteins[10, 16, 20] as well as the cytotoxic necrotizing factor type [21]. Moreover, in association with laminin-1, LRP/LR is involved in crucial cellular processes such as cell adhesion, migration, proliferation and differentiation[22]. However, since this receptor is overexpressed in cancer cells these processes are augmented and contribute to cellular transformation, which describes the role of LRP/LR in tumor invasion and metastasis. A direct correlation between high levels of LRP/LR expression and tumor aggressiveness has been noted in numerous cancers, including, fibrosarcoma[23], breast[24], cervical[25], colon[26], lung[27], prostate[28], oesophageal[29], liver[30], gastric[31] and ovarian[32] cancer. However, incubation of the above mentioned metastatic cancers with anti-LRP/LR specific antibody IgG1-iS18 resulted in significant impediment of adhesion and invasion, the two key steps of metastasis [29, 30, 33]. Furthermore, our laboratory recently illustrated that LRP/LR plays a role in another eminent hallmark of cancer, angiogenesis, as treatment of blood vessels formed *in vitro* with an anti-LRP/LR specific antibody W3 significantly hampered blood vessel formation [34].

Cancerous cells strive to circumvent cell death and the elevated levels of LRP/LR also assist cancer cells in this regard by associating with the Midkine protein and connect the nuclear envelope and chromatin during interphase in order to retain chromosomal stability and in turn maintaining cell viability[35]. Our laboratory exemplified that LRP/LR indeed plays a role in maintenance of cellular viability as downregulation of LRP/LR with specific siRNAs resulted in a significant reduction in survival of cervical and lung cancer cells as a consequence of apoptosis induction[36].

This affiliation between LRP/LR expression and tumor aggressiveness proposes LRP/LR as a promising tool for cancer treatment and since the targeting of LRP/LR on cancerous cells has proven successful with regards to reduction of tumor metastasis[29, 30, 33, 37] and hampering of angiogenesis induction[34], exploring the role of LRP/LR on the viability of cancerous cells has gained huge interest. Therefore, this study was aimed at elucidating the effect of siRNA-mediated knockdown of LRP/LR on the cellular viability of breast and oesophageal cancers which are ranked the second and eighth most commonly diagnosed cancers worldwide (GLOBOCAN 2012). These high rankings indicate the importance of finding alternative therapeutic options and although a similar study has been carried out in our laboratory on lung and cervical cancer cells it is noteworthy to comprehend that not all types of cancers will be responsive to siRNA treatment. Therefore it is imperative that similar studies are carried out on other cancer types in order to elucidate whether or not targeting of LRP/LR with siRNAs may be used as an alternative broad spectrum therapeutic tool for the treatment of cancer.

This study has illustrated significant reduction in viability of breast MCF-7, MDA-MB 231 and oesophageal WHCO1 cancer cells after significant downregulation of LRP/LR. Furthermore, the observed cell death is due to the induction of apoptosis, indicated by phosphatidylserine exposure and prominent nuclear morphological changes observed in the above mentioned breast and oesophageal cancer cells.

## Materials and Methods

### Cells and cell culture conditions

Human breast adenocarcinoma MCF-7 cells were cultured in Dulbecco modified eagle’s medium (DMEM) high glucose (4.5 g/l) (Invitrogen Gibco). Breast cancer cells MDA-MB 231 and WHCO1 oesophageal cancer cells were cultured in DMEM/ Ham’s-F12 (1:1). All media was supplemented with 10% fetal calf serum (FCS) and 1% penicillin/streptomycin and cell lines were grown in humidified incubator at 37°C and 5% CO_2_.

MCF-7 and MDA-MB 231 breast cancer cells were commercially obtained from the American Tissue Culture Collection (ATCC) with catalogue numbers ATCC^®^HTB22™ and ATCC^®^HTB26™, respectively.

The WHCO1 oesophageal cancer cell line is not commercially available; it was propagated *in vitro* from an epithelial cell line of tumor biopsy material of a patient with this type of oesophageal carcinoma [29, 38].

### siRNA-mediated downregulation of LRP

All cell lines were seeded in 6-well plates and, after 24 h incubation, transfected for LRP knockdown with siRNA-LAMR1 and siRNA-scr was used as a control. MDA-MB 231 cells were also transfected with esiRNA-RPSA (Sigma) for knockdown of LRP and esiRNA-RLUC (Sigma) was used as a control. The siRNA sequences as well as transfection reagents used are provided in Table 1. Post72 h incubation (24 h, 48 h and 72 h for MDA-MB 231 cells) in a humidified incubator set at 37°C and 5% CO_2,_ cells were lysed and lysates were analysed by western blotting.

**Table 1:** Sequence of control siRNA-RLUC as well as siRNA used for downregulation of LRP/LR.

siRNA	Sequence	Transfection reagent
siRNA-LAMR1	UAUCAUAAAUCUCAAGAGG	Transfection reagent 1 (Dharmacon)
siRNA-scr	ON-TARGETplus Non-targeting Control siRNAs #4, D-001810-04-05 (Dharmacon)	Transfection reagent 1 (Dharmacon)
esiRNA-RPSA	CCTCTCACGGAGGCATCTTATGTTAACCTACCTACCATTGCGCTGTGTAACACAGATTCTCCTCTGCGCTATGTGGACATTGCCATCCCATGCAACAACAAGGGAGCTCACTCAGTGGGTTTGATGTGGTGGATGCTGGCTCGGGAAGTTCTGCGCATGCGTGGCACCATTTCCCGTGAACACCCATGGGAGGTCATGCCTGATCTGTACTTCTACAGAGATCCTGAAGAGATTGAAAAAGAAGAGCAGGCTGCTGCTGAGAAGGCAGTGACCAAGGAGGAATTTCAGGGTGAATGGACTGCTCCCGCTCCTGAGTTCACTGCTACTCAGCCTGAGGTTGCAGACTGGTCTGAAGGTGTACAGGTGCCCTCTGTGCCTATTCAGCAATTCCCTACTGAAGACTGGAGCG	Lipofectamine 3000
esiRNA-RLUC	GATAACTGGTCCGCAGTGGTGGGCCAGATGTAAACAAATGAATGTTCTTGATTCATTTATTAATTATTATGATTCAGAAAAACATGCAGAAAATGCTGTTATTTTTTTACATGGTAACGCGGCCTCTTCTTATTTATGGCGACATGTTGTGCCACATATTGAGCCAGTAGCGCGGTGTATTATACCAGACCTTATTGGTATGGGCAAATCAGGCAAATCTGGTAATGGTTCTTATAGGTTACTTGATCATTACAAATATCTTACTGCATGGTTTGAACTTCTTAATTTACCAAAGAAGATCATTTTTGTCGGCCATGATTGGGGTGCTTGTTTGGCATTTCATTATAGCTATGAGCATCAAGATAAGATCAAAGCAATAGTTCACGCTGAAAGTGTAGTAGATGTGATTGAATCATGGGATGAATGG	Lipofectamine 3000

### SDS-PAGE and Western blotting

Total LRP levels were analysed by western blotting. Approximately 10 μg of total protein was separated on a 12% gel using the sodium dodecyl sulphate polyacrylamide gel electrophoresis (SDS-PAGE) system and the resulting protein pattern was transferred onto a polyvinylidine fluoride (PVDF) membrane using a 1 x transfer buffer at 450 mV for 45 minutes. Resulting protein membrane was blocked with 3% BSA in 0.1% PBS Tween to prevent non-specific binding of the primary antibody IgG1-iS18 (1:10 0000) for the detection of LRP. After 1 h incubation with the primary antibody, the membrane was rinsed three times with PBS Tween and incubated for 1 h with goat anti-human peroxidise labelled secondary antibody (1:5 000). The membrane was washed again with PBS Tween, an enhanced chemillumniscent substrate (Thermoscientific) was used for detection of the HRP and the resulting chemilluminescence was developed and fixed onto an X-ray film in the dark or detected with the Chemidoc system (Biorad).

### MTT assay

MCF-7, MDA-MB 231 and WHCO1 cancer cells were seeded in a 24-well plate transfected when a confluency of 80% was reached and allowed to incubate in the incubator at 37°C with 95% air and 5% CO_2_ for 72 h (or 24 h, 48 h and 72 h after transfection of MDA-MB 231 cells). Cells treated with 8 mM PCA (protocatechuic acid) was used as a positive control. MTT (3-(4, 5-dimethylthiazol-2-yl)-2-5-diphenyltetazolium bromide) is a yellow tetrazolium salt that is converted into purple formazan crystals by the mitochondrial succinate dehydrogenase enzyme active in mitochondria of viable cells thus providing an estimation of cellular viability. A 1 mg/ml solution of MTT dissolved in PBS was added in each well and allowed to incubate for 2 h. Media was discarded, the resulting formazan crystals were dissolved in 500 μl of DMSO and absorbance of each well was measured at 570 nm using an ELISA reader.

### Annexin V-FITC /7-AAD assay

MCF-7, MDA-MB 231 and WHCO1 cancer cells were seeded in 24-well plates, transfected at 80% confluency and incubated for 72 h as described above. Cells treated with 8 mM PCA served as a positive control. Cells were harvested using Trypsin/EDTA and resulting cell suspensions were washed with ice-cold PBS and centrifuged for 5 minutes at 4°C at 500 x g. According to the manufacturers’ instructions (Beckman Coulter, Immunotech, Marseille, France), supernatants were discarded and pellets were resuspended in 100 μl of Binding buffer whilst working on ice. Annexin V-FITC solution and 7-AAD viability dye at 10 μl and 20 μl respectively was added to the 100 μl cell suspensions. Cell suspensions were incubated on ice in the dark for 15 minutes and 200 μl of ice-cold Binding buffer was added. Cell populations were analyzed using the BD Accuri flow cytometer and Annexin V-FITC was measured at an excitation wavelength of 495 nm, emission of 519 nm and 7-AAD at excitation of 488 nm and emission at 640 nm. BD Accuri C6 software was used for data analysis. A total of 10^4^ events were recorded for each sample and untreated control cells were used to set gates.

### Immunofluorescence microscopy

This technique was employed in order to analyse the nuclear morphological alterations that occur in the cell upon cell death induction.

MCF-7, MDA-MB 231 and WHCO1 cancer cells were seeded onto coverslips in wells of a 6-well plate and allowed to reach 80% confluency in a 5% CO_2_ humidified incubator set at 37°C. Cells were transfected as described above and cells treated with 8 mM PCA served as an apoptosis control. Post 72 h incubation media was aspirated from wells and cells were fixed in 4% paraformaldehyde (PFA) followed by several washes with PBS. Hoescht 33324 stain diluted in PBS (137 mM NaCl, 12 mM Phosphate, 2.7 mM KCl, pH 7.4) was administered for 5–10 minutes to allow for staining of the nucleus and cells were then mounted onto a clean slide using GelMount (Sigma-Aldrich) for 1 h in the dark.

### Statistical evaluations

Densitometry analysis for LRP/LR was performed by normalising to the loading control and is reported as a percentage compared to the untreated control (n = 3).

Nuclear morphological changes were quantified with ImageJ software and are represented as the average nuclear area.

Data was statistically analyzed using the Student’s t-test with a confidence interval of 95% with p-values of less than 0.05 considered significant.

## Results

### Knockdown of LRP expression in breast and oesophageal cancer cells

**Fig 1:**
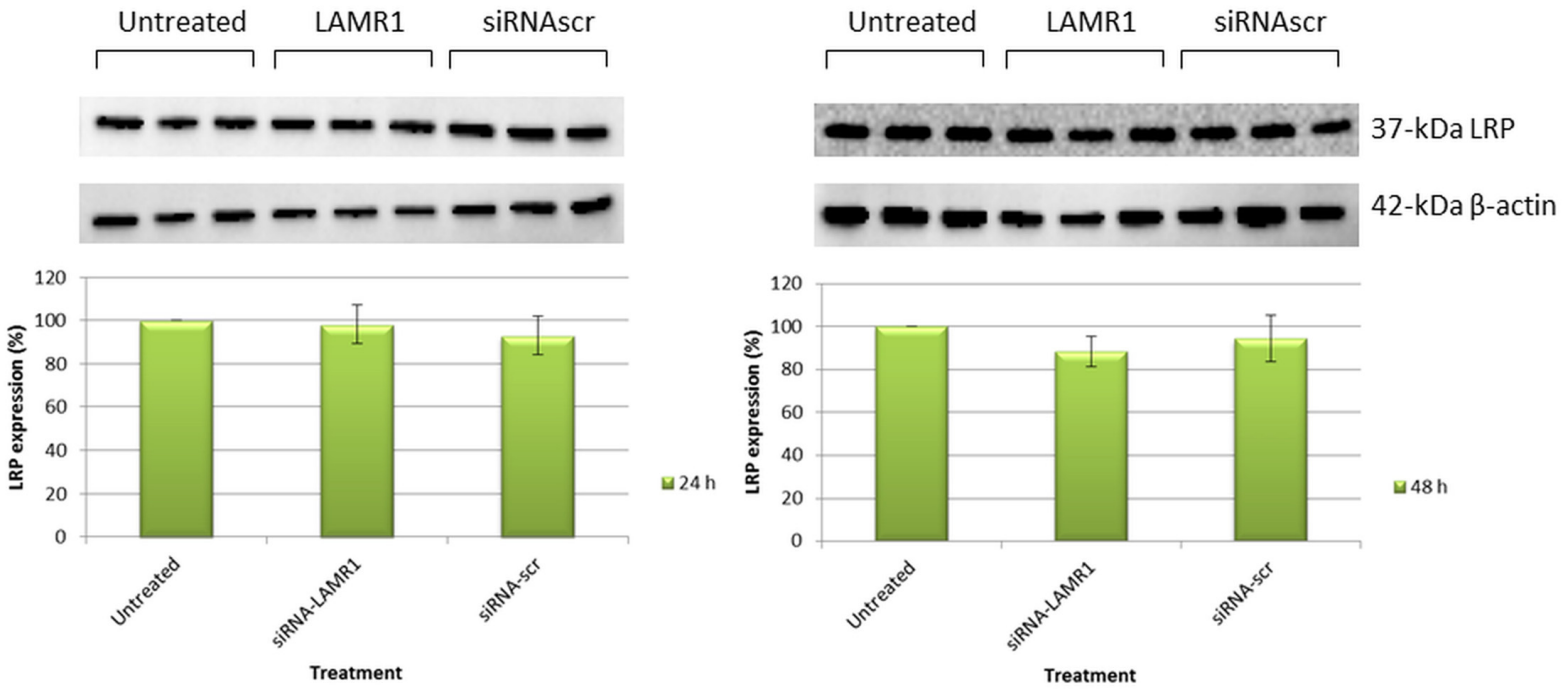
Western blot analysis of LRP expression in MDA-MB 231 cells 24 h and 48 h post-transfection with siRNA-LAMR1. No significant decrease in expression of the 37-kDa LRP was observed 24 h and 48 h after transfection when compared to non-transfected cells. The standard deviation is represented by error bars (n = 3).

**Fig 2:**
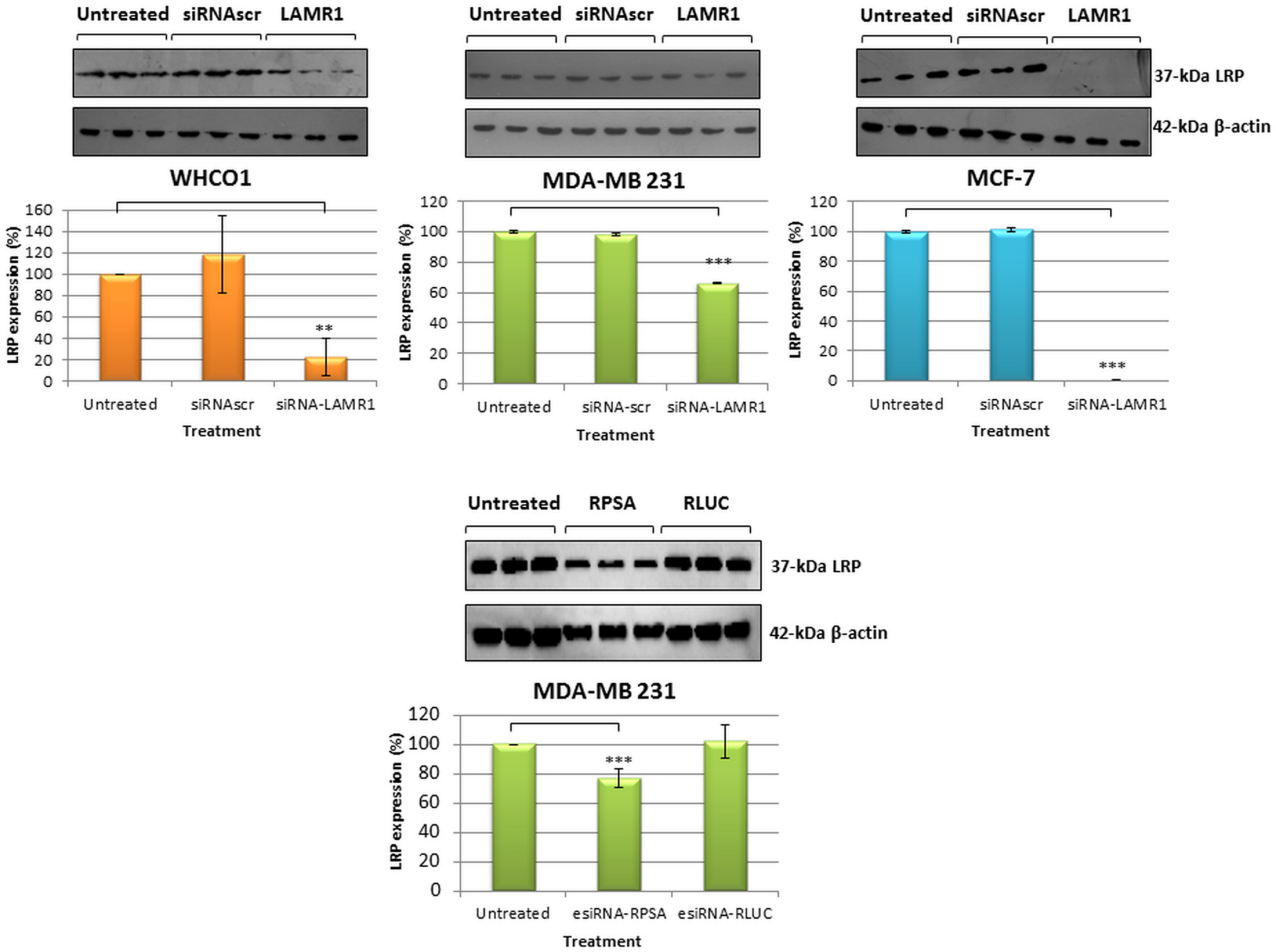
LRP expression in WHCO1, MDA-MB 231 and MCF-7 cells 72 h post-transfection. WHCO1 and MCF-7 cells were transfected with siRNA-LAMR1 and MDA-MB 231 cells with siRNA-LAMR1 and esiRNA RPSA. Densitometric analysis of the western blot signals revealed significant (p < 0.05, n = 3) differences in LRP expression inWHCO1, MDA-MB 231 and MCF-7 cells, respectively (compared to non-transfected cells). Error bars represent standard deviation.

High expression of LRP/LR tends to augment the aggressive behaviour of cancer cells by mediating metastasis, tumor angiogenesis and the ability to evade cell death [29, 30, 33, 34, 36]. The MDA-MB 231 breast and WHCO1 oesophageal cancer cells lines were no exception as Khumalo *et al*. illustrated enhanced metastatic ability accompanied by high expression levels of LRP/LR[29]. The results of this study paved way for the investigation of how the knockdown of the expression of LRP/LR would impact on the cellular viability of these cell lines as well as the non-metastatic MCF-7 breast cancer cell line. We used siRNA LAMR1 as well as esiRNA RPSA (MDA-MB 231 cells) to downregulate LRP by targeting the messenger RNA of LRP (siRNA-scr and esiRNA RLUC acted as negative controls). Densitometry analysis of the western blots revealed that no decrease in LRP was observed 24 h or 48 h after transfection of MDA-MB 231 cells with siRNA LAMR1 (Fig 1). However there was a significant decrease in LRP levels 72 h after transfection of WHCO1, MDA-MB 231 and MCF-7 cells (Fig 2). A 78%, 34% and 100% decrease in LRP expression was observed for the respective cell lines when compared to non-transfected negative controls set to a 100%. Western blot analysis of MDA-MB-231 cells transfected with esiRNA-RPSA showed a 26% downregulation of LRP (Fig 2).

### siRNA-mediated downregulation of LRP causes significant decrease in viability of breast and oesophageal cancer cells

**Fig 3:**
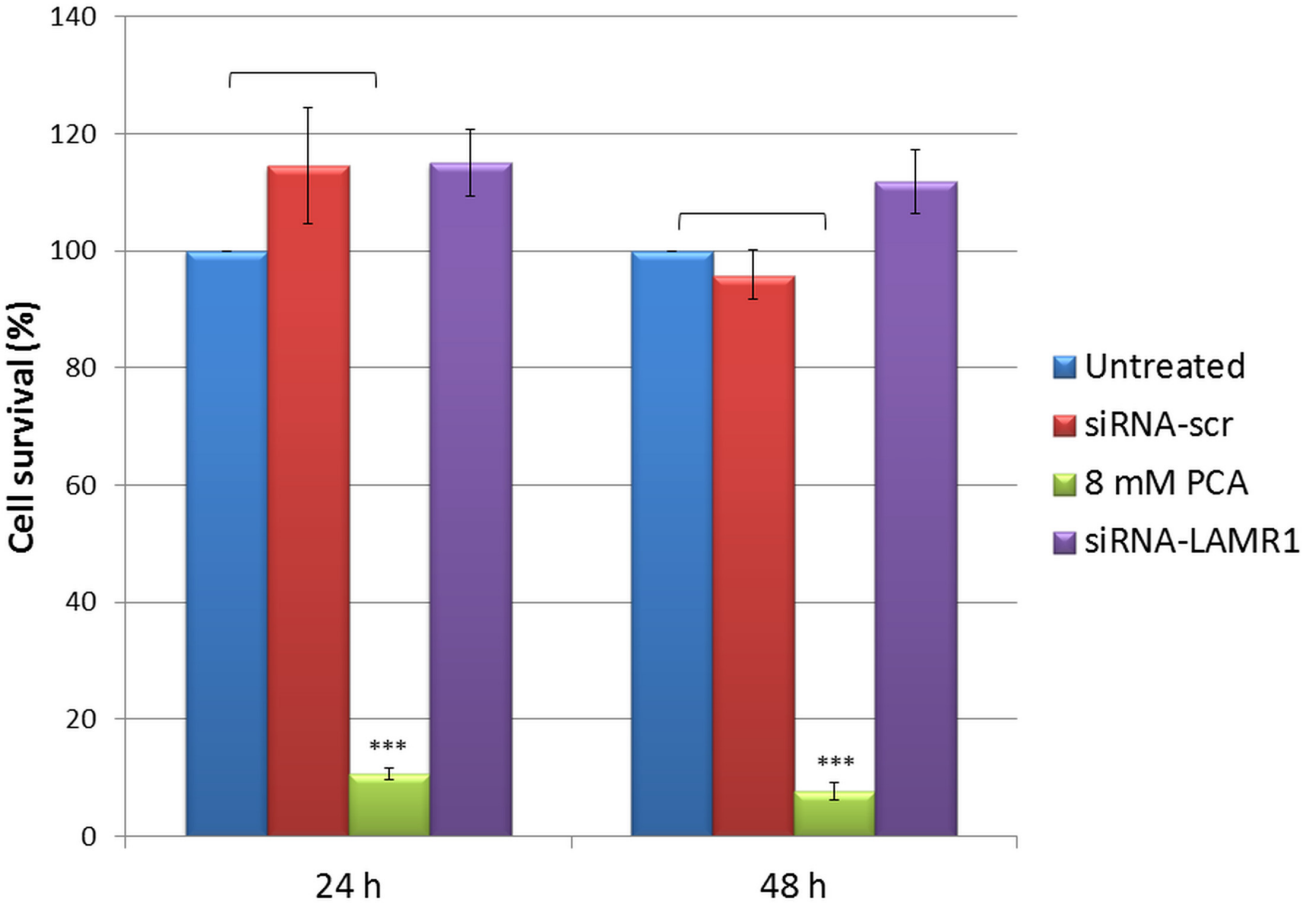
Cell viability of MDA-MB 231 cells 24 h and 48 h after downregulation of LRP. MDA-MB 231 cells were transfected with siRNA-LAMR1 and cell viability was determined with the MTT assay. PCA (8 mM) and siRNA-scr were used as positive and negative controls, respectively. The viability was compared to the untreated control set at 100%. Error bars indicate standard deviation (n = 3).

**Fig 4:**
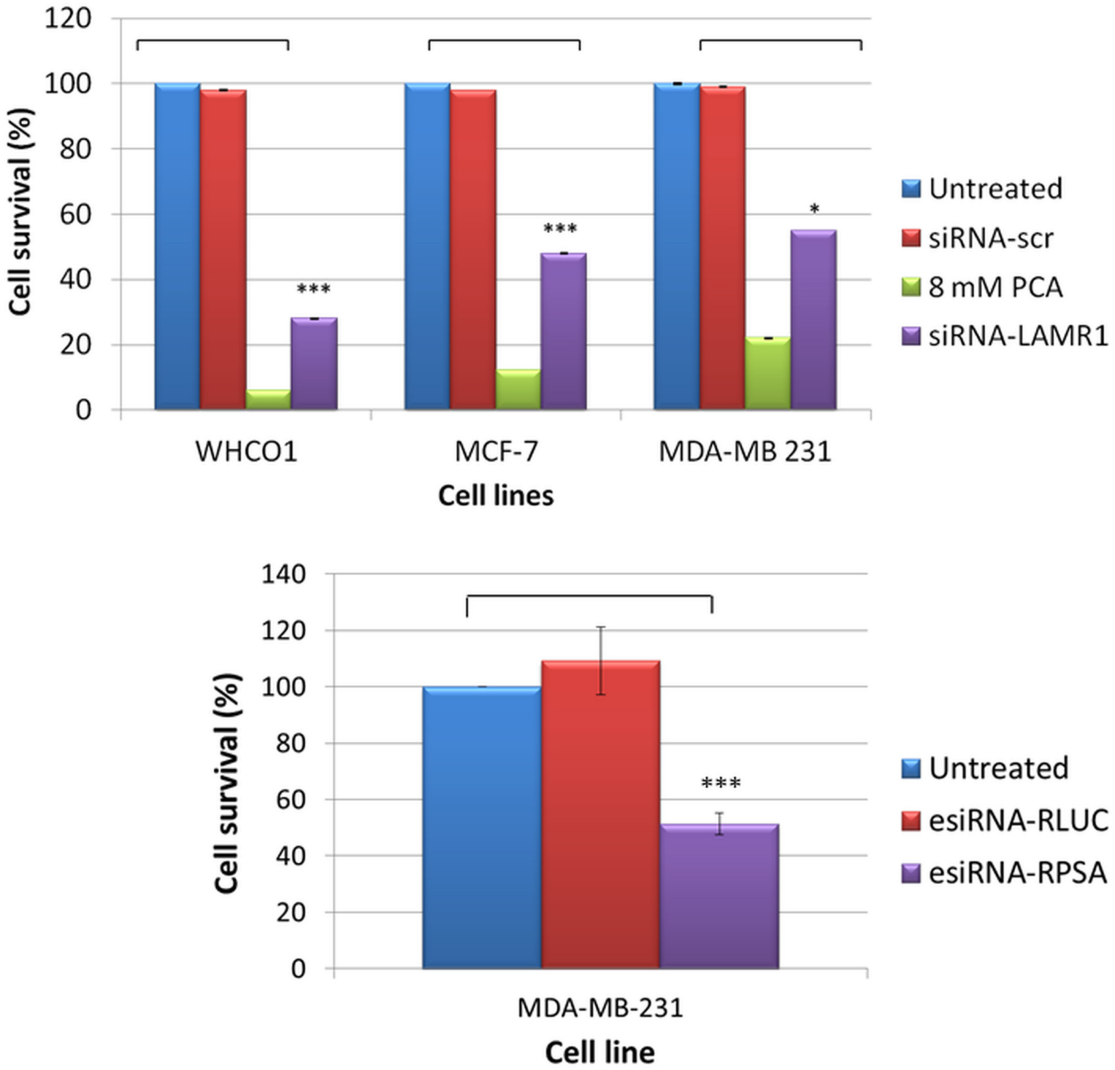
The effect of siRNA-mediated downregulation of LRP expression on the cellular viability of WHCO1, MCF-7 and MDA-MB 231 cells. The viability of WHCO1, MCF-7 and MDA-MB 231 cells was analysed 72 h post-transfection using an MTT assay. siRNA-LAMR1 treated WHCO1, MCF-7 and MDA-MB 231 as well as esiRNA-RPSA treated MDA-MB 231 cells revealed a significant reduction in cellular viability compared to untreated cells set to 100%. 8mM PCA and siRNA-scr were used as positive and negative controls, respectively. The error bars represent the standard deviation (n = 3) and a significant difference (* p < 0.05, ** p < 0.01, *** p <0.001) between the untreated control and the treated samples is indicated by an asterisk.

In this study the MTT results suggest that LRP/LR plays a role in the viability of cancer cells. No significant decrease in viability of MDA-MB-231 was observed 24 h or 48 h after transfection (Fig 3), however the cell viability of the MCF-7, MDA-MB 231 and WHCO1 cancer cells was significantly decreased by 52%, 45% and 72%, respectively (Fig 4) 72 h following the downregulation of LRP/LR expression. 8mM PCA (a known apoptosis-inducer) was used as a positive control and cells transfected with the non-targeting siRNA-scr (or esiRNA-RLUC) posed as a negative control. A significant decrease in cellular viability was also noted in the 8mM PCA treated cells and the non-targeting siRNAs had no effect (Fig 4).

### Knockdown of LRP induces apoptosis of breast and oesophageal cancer cells

**Fig 5:**
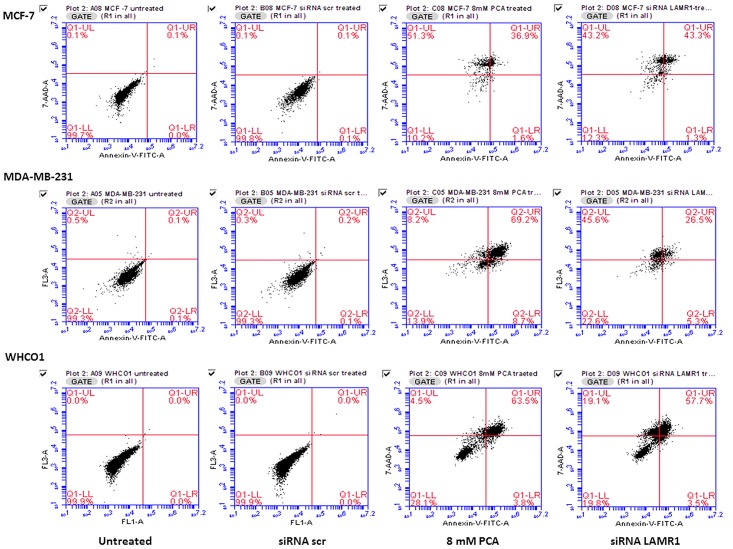
The effect of siRNA-mediated downregulation of LRP expression on cell membrane integrity. 72 h post-transfection, MCF-7, MDA-MB 231 and WHCO1 cells were stained with Annexin-V FITC and 7-AAD then analysed by flow cytometry. siRNA-LAMR1 treated MCF-7, MDA-MB 231 and WHCO1 cells revealed high FITC and 7-AAD signals indicative of cells undergoing early and late apoptosis, compared to untreated and siRNA-scr treated cells (8mM PCA was used as a positive control).

To elucidate a possible mechanism for the decrease in cellular viability upon knockdown of LRP in MCF-7, MDA-MB 231 and WHCO1 cancer cells, changes in the integrity of cell membrane and nuclear morphology were evaluated. Cells that were transfected with siRNA-LAMR1, siRNA-scr (negative control) and 8mM PCA (positive control) as previously described were labelled with Annexin V-FITC and 7-AAD. During apoptosis induction, cell integrity is lost and phospholipids such as phosphatidylserine (PS) become exposed at the cell surface. Annexin-V binds to PS with very high affinity thus labelling cells undergoing apoptosis when membrane blebbing occurs. 7-AAD distinguishes between early and late apoptotic cells. Indeed, the reduced cellular viability of breast MCF-7 and oesophageal WHCO1 cancer cells was due to the induction of apoptosis as depicted by the shift of cells from the bottom left quadrant (live cells) to the bottom right and top right quadrants (representing early and late apoptotic cells, respectively) (Fig 5). The MDA-MB 231 cells were observed to be undergoing late apoptosis as well as necrosis (Fig 5) after the knockdown of LRP expression.

**Fig 6:**
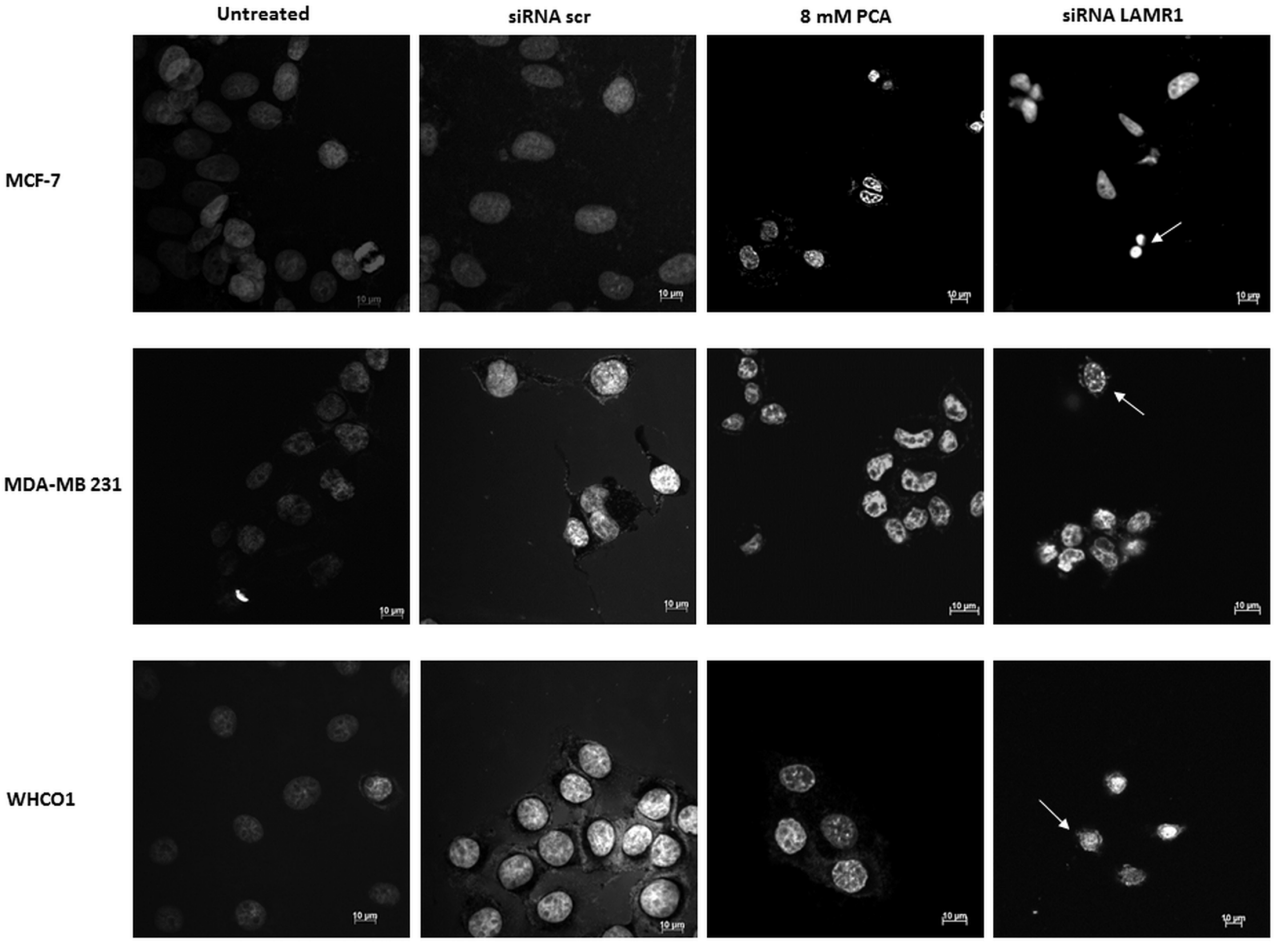
The effect of siRNA-mediated downregulation of LRP expression on nuclear morphology. 72 h post-transfection, MCF-7, MDA-MB 231 and WHCO1 cells were stained with Hoescht 33324 and viewed by immunofluorescence microscopy. siRNA-LAMR1 treated MCF-7, MDA-MB 231 and WHCO1 cells displayed condensed nuclei and decreased nuclear integrity (indicated by white arrows), compared to untreated and siRNA-scr treated cells (8mM PCA was used as a positive control).

**Fig 7:**
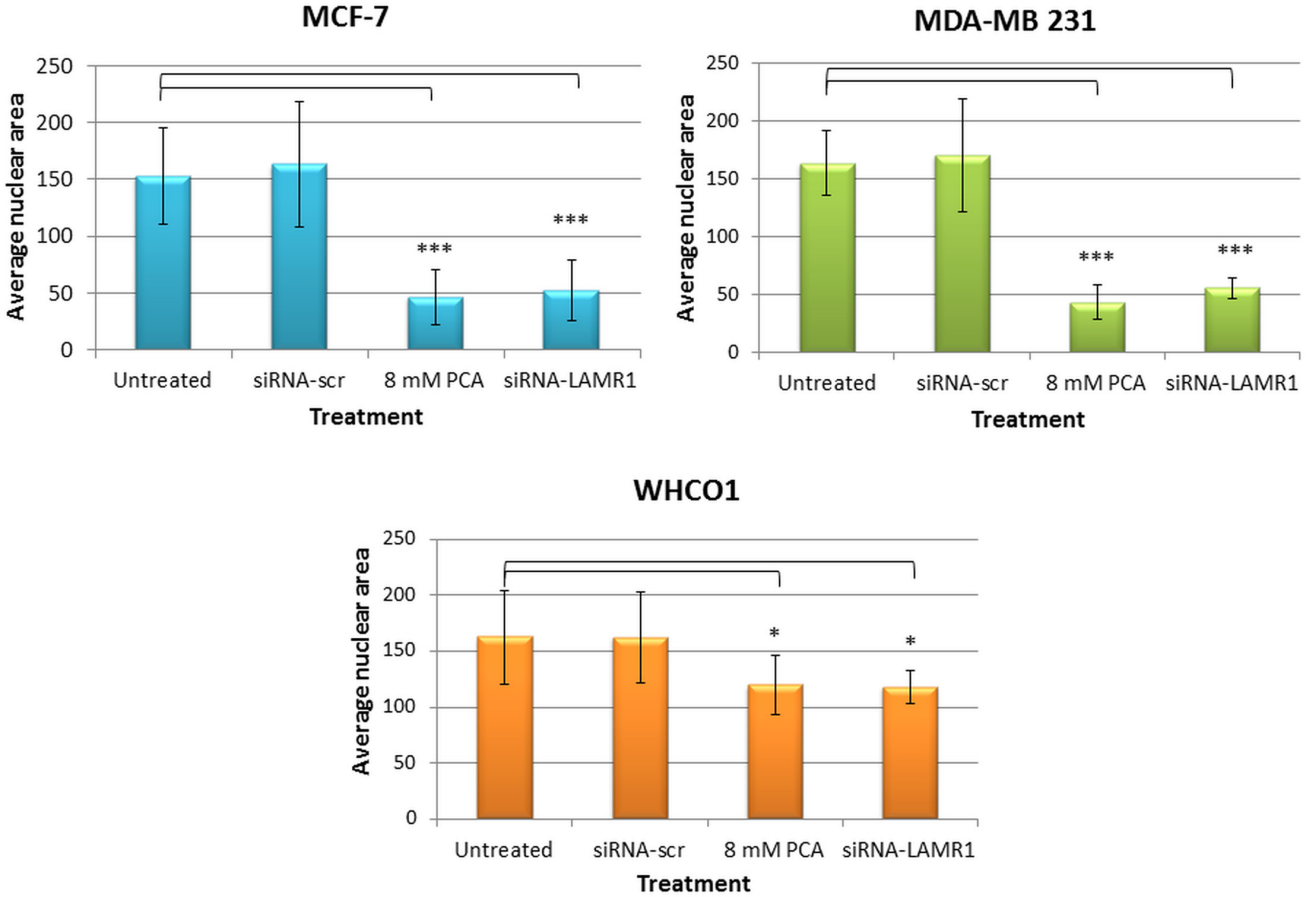
Changes in nuclear area after siRNA-mediated downregulation of LRP. 72 h post-transfection, MCF-7, MDA-MB 231 and WHCO1 cells were stained with Hoescht 33324 and viewed by immunofluorescence microscopy. The change in nuclear morphology was quantified with ImageJ software and is represented as the average nuclear area. The error bars indicate the standard deviation and a significant difference (* p < 0.05, ** p < 0.01, *** p <0.001) between the untreated control and the treated samples is shown by an asterisk.

In addition to the observation of this fundamental hallmark of apoptosis, MCF-7, MDA-MB 231 and WHCO1 cells, transfected with siRNA-LAMR1 and stained with the fluorescent nuclear dye Hoechst 33324 exhibited nuclei constriction, loss of nuclear morphology and integrity when compared to siRNA-scr-transfected cells as indicated by the white arrows (Fig 6). A loss of nuclear morphology was exhibited by the crescent-shaped nuclei that are due to the collapse of chromatin as clearly observed in the 8mM PCA and siRNA-LAMR1 treated cell micrographs of MDA-MB 231 cells. siRNA-LAMR1 treated MCF-7 cells exhibited shrinked nuclei that form small balls that form apoptotic bodies whereas a majority of siRNA-LAMR1 treated WHCO1 cells underwent gradual chromatin condensation (Fig 6). The changes in nuclear morphology were quantified by measuring the nuclear area of the cells. Treatment of MCF-7, MDA-MB 231 and WHCO1 cells with 8 mm PCA as well as siRNA-LAMR1 furthermore caused a significant decrease in nuclear area as shown in Fig 7.

## Discussion

The role of LRP/LR in cancer progression has been profoundly studied, with numerous studies suggesting that the laminin receptor plays a prominent role in mediating some cancer hallmarks. The role of this receptor in these processes is anticipated because of its numerous cellular localisations i.e. the cell surface, nucleus, perinuclear compartment and cytosol thus mediating crucial physiological processes. However the high levels of LRP expression in cancer cells allow these cells to override these processes for survival purposes[3].

Metastatic breast MDA-MB231 and oesophageal WHCO1 cancer cells, the main focus of this study express high levels of LRP/LR on the cell surface and in the cell as determined in a previous study[29]. The non-metastatic MCF-7 breast cancer cell line was observed to express low LRP/LR levels on the cell surface [30] and was included in this study to investigate the difference between metastatic and non-metastatic cells regarding the effect of knocking down LRP/LR. In order to investigate the role of LRP/LR in cell viability, and apoptosis, we knocked down LRP expression by RNA interference. Indeed, LRP downregulation was obtained 72 h after transfection with siRNA-LAMR1 in all cell lines and additionally with esiRNA-RPSA in MDA-MB 231 cells as confirmed by western blotting and densitometry analysis. No significant reduction in expression of LRP was observed 24 h and 48 h after transfection of MDA-MB 231 cells (Fig 3). The expression levels of LRP were reduced by 34% in MDA-MB 231 (26% with esiRNA-RPSA), 78% in WHCO1 and 100% in MCF-7 cancer cells (Fig 2).

The evasion of apoptosis is a fundamental hallmark of cancer cells hence the effect of knockdown of LRP on the viability of the above mentioned breast and oesophageal cancer cell lines was investigated. No decrease in expression of LRP or decrease in viability of MDA-MB 231 cells was observed 24 h and 48 h after transfection (Figs 1 and 3). However, although a 100% knockdown of LRP was observed in MCF-7 breast cancer cells 72 h after transfection, only a 52% reduction in cellular viability was noted whereas the decrease in viability of the WHCO1 and MDA-MB 231 cells correlated to the reduction in LRP expression (Figs 2 and 4). This may suggest that the anti-apoptotic role of LRP/LR varies with respect to cell line. It is hypothesized that LRP/LR associates with the nuclear envelope and chromatin during interphase, connecting the latter to the former thus retaining chromosomal stability and in turn maintaining cellular viability. LRP/LR accomplishes this by binding to the growth factor Midkine, which is known to promote cell migration, proliferation and survival by inducing nuclear localization [35]. Knockdown of LRP results in cell death of breast MCF-7, MDA-MB 231 and oesophageal WHCO1 cancer cells, which could be due to autophagy, apoptosis or necrosis. Sustanad and Smith illustrated that silencing of LRP/LR induces apoptosis in Hep3B cells; therefore we employed apoptotic assays to elucidate whether apoptosis is indeed the mode of cell death impeded by LRP/LR in these breast and oesophageal cancer cell lines[37]. We focused on the prominent hallmarks of apoptosis; changes in the cell membrane integrity as well as alterations in the nuclear morphology of the cells [39, 40]. Post-transfection with siRNA-LAMR1 and treatment with 8mM PCA, breast MCF-7, MDA-MB 231 and oesophageal WHCO1 cancer cells labelled with Annexin V-FITC/7-AAD resulted in a shift of the majority of cells to the quadrants displaying late apoptotic and necrotic cells (Fig 5). The untreated and siRNA-scr (negative control) populations of these cell lines displayed low FITC and 7-AAD signals in the bottom left quadrant (viable cells) (Fig 5).

The high FITC and 7-AAD signals observed in siRNA-LAMR1-treated breast and oesophageal cancer cell lines indicate the loss of cell membrane integrity and asymmetry of the membrane phospholipids. Annexin V binds to the phospholipid, phosphatidylserine (PS) that localises on the inner cell membrane, however, during apoptosis induction, PS becomes exposed at the cell surface hence the high Annexin-V FITC signal [41, 42]. This exposure of PS is crucial as it is a signal required for the recognition and removal of apoptotic cells by lysozymes or neighbouring phagocytic cells, thus not posing harmful consequences to other cells and the cellular environment[43]. Moreover, 7-AAD binds the DNA guanine-cytosine base pair allowing for distinction between early or late apoptotic and necrotic cells; thus the high 7-AAD signal was indicative of late apoptotic and necrotic cells in the populations of breast MCF-7, MDA-MB 231 and oesophageal WHCO1 cancer cells (Fig 5). It was observed that a majority of MCF-7 and WHCO1 cells were at a late stage of apoptosis whereas a majority of MDA-MB 231 cells experienced necrosis after downregulation of LRP/LR. It is important to note that the Annexin V-FITC/7-AAD experiments were carried out 72 h post transfection with siRNA-LAMR1 and analysis after shorter incubation times could yield different results.

As mentioned before, the role of LRP in the maintenance of cellular viability has been reported in previous studies [35–37] and this study confirmed that LRP does indeed play a crucial role in maintaining cell viability and upon knockdown of this protein apoptosis was induced. LRP also localises in the perinuclear compartment and nucleus [12, 13], therefore we investigated whether knockdown of this receptor affected the integrity of the nucleus. Indeed, post transfection with siRNA-LAMR1 morphological changes were observed in MCF-7, MDA-MB 231 and WHCO1 cancer cells (compared to untreated and siRNA-scr-treated cells); the nuclei appeared constricted and a decrease in nuclear area was observed (Figs 6 and 7). Both the loss of membrane integrity and change in nuclear morphology observed in these cell lines are indicative of apoptosis occurrence[40], thus suggesting that LRP maintains cellular viability by impeding apoptosis.

The pathological potential of LRP/LR in cancer has been targeted using numerous anti-LRP/LR tools including anti-LRP/LR specific antibodies in order to impede cancer propagating processes including, increased invasion[3], cellular proliferation[44], metastasis[29, 33] and evasion of apoptosis[36]. Therefore, targeting of LRP/LR poses as a promising alternative therapeutic approach for the treatment of cancer by hampering the occurrence of the above mentioned cancer promoting processes. The fact that downregulation of LRP in cervical and lung cancer cells resulted in induction of apoptosis [36] does not implicate that LRP knockdown has the same effect in other cancer types. Therefore, we investigate the effect of LRP knockdown in breast and oesophageal cancer cells, major deleterious cancer types worldwide, in this study. We proved that LRP knockdown in these cancer cell lines also promotes cell death by predominantly late apoptosis induction, although the knockdown in metastatic MDA-MB 231 breast cancer cells also induces necrosis. However, it must be stressed that the laminin receptor is imperative for physiological processes in normal cells such as cell attachment, differentiation, motility, thus its inhibition may cause detrimental effects that can compromise the health of the individual, although knockdown of LRP in the brain by antisense RNA technology did not result in any physiological side effects in mice[45]. Consequently, studies concentrating on potentially appropriate gene delivery systems such as lentiviral or adeno-associated viral systems for siRNA targeting LRP/LR are crucial and with successful animal trials employing application of siRNAs targeting LRP could be deemed as a potential therapeutic approach for the treatment of breast and oesophageal cancers.

## Abbreviations

β: beta
DMEM: Dulbecco’s Modified Eagle Medium
EDTA: ethylenediaminetertraacetic acid
FCS: fetal calf serum
FITC: fluorescein isothiocynate
IgG: immunoglobulin
kDa: kilodalton
LRP/LR: laminin receptor precursor/ laminin receptor
PFA: paraformaldehyde
PBS: phosphate based saline
SDS: sodium dodecyl sulphate
PVDF: polyvinylidine fluoride
HRP: horse radish peroxidise
DMSO: dimethyl sulfoxide
